# Integrative Phylogeography of *Calotriton* Newts (Amphibia, Salamandridae), with Special Remarks on the Conservation of the Endangered Montseny Brook Newt (*Calotriton arnoldi*)

**DOI:** 10.1371/journal.pone.0062542

**Published:** 2013-06-04

**Authors:** Emilio Valbuena-Ureña, Fèlix Amat, Salvador Carranza

**Affiliations:** 1 Unitat de Zoologia (Facultat de Biociències), Universitat Autònoma de Barcelona, Cerdanyola del Vallès, Catalonia, Spain; 2 Centre de Fauna Salvatge de Torreferrussa, Forestal Catalana, Barcelona, Catalonia, Spain; 3 Museu de Ciències Naturals de Granollers, Àrea d'Herpetologia, Granollers, Catalonia, Spain; 4 Institute of Evolutionary Biology (CSIC-Universitat Pompeu Fabra), Barcelona, Catalonia, Spain; Institut de Biologia Evolutiva – Universitat Pompeu Fabra, Spain

## Abstract

The genus *Calotriton* includes two species of newts highly adapted to live in cold and fast-flowing mountain springs. The Pyrenean brook newt (*Calotriton asper*), restricted to the Pyrenean region, and the Montseny brook newt (*Calotriton arnoldi*), endemic to the Montseny massif and one of the most endangered amphibian species in Europe. In the present manuscript, we use an integrative approach including species distribution modeling (SDM), molecular analyses of mitochondrial and nuclear DNA sequence data and morphology to unravel the historical processes that have contributed to shaping the biogeography and genetic structure of the genus *Calotriton*, with special emphasis on the conservation of *C. arnoldi*. The results of the molecular analyses confirm that, despite having originated recently, being ecologically similar and geographically very close, there is no signal of hybridization between *C. asper* and *C. arnoldi*. SDM results suggest that tough environmental conditions on mountains tops during glacial periods, together with subsequent warmer periods could have prevented the contact between the two species. Within the critically endangered *C. arnoldi*, a high genetic structure is revealed despite its extremely small distribution range compared to *C. asper*. Haplotype networks, AMOVA and SAMOVA analyses suggest that two distinct groups of populations can be clearly differentiated with absence of gene flow. This is in concordance with morphological differentiation and correlates with its geographical distribution, as the two groups are situated on the eastern and western sides of a river valley that acts as a barrier. The genetic and morphological results are highly important for the ongoing conservation program of *C. arnoldi* and strongly justify the management of this species into at least two independent evolutionary significant units (eastern and western sectors) to guarantee the long-term population viability.

## Introduction

The evolutionary history of species strongly depends on the variation of their distributions throughout time and space. For instance, it is well known that during the Pleistocene many species shifted their distribution ranges as a result of the climatic cycles ([1] and references herein). The Quaternary age includes the last 2.6 My encompassing the Pleistocene and Holocene periods to the present. The Holocene is characterized for its relative climatic stability compared to the Pleistocene. The latter is notable for its strong oscillations in the climate with subsequent glacial and interglacial periods that have had a great impact on the distribution and evolution of species [2]. The effect of the ice ages on European species has been extensively studied ([3] and references herein). The fluctuating environmental conditions found during the recurrent ice ages, forced populations to migratory processes of contraction and expansion of their geographic ranges. During cold periods, the mountainous parts of southern Europe are likely to have provided suitable habitats for the species to survive acting as glacial refugia [4]. Further expansion from refugia occurred when the temperature increased. These successive colonization processes implied bottlenecks that may facilitate allopatric speciation in refugia [5], [6], [7]. Genetic divergence among populations may occur during periods of isolation, while dispersion processes and gene flow may take place during connectivity periods [4], [8], [9], [10]. Therefore, the actual distribution and genetic characteristics of species have been influenced by their past population history trends [11], [12].

Amphibians are good models to explore the influence of historical aspects on the genetic structure at different geographic scales ([13], [14], [15], [16], among others). This can be explained by the retention of strong phylogeographic signal due to the low dispersal capacity [17], and sensibility to small environmental changes [6]. Population differentiation related to the Pleistocene glaciations has been postulated among alpine urodeles [18], [19], [20]. Species distribution models (SDM) are useful for inferring climate-based potential distributions throughout the recent geological history and testing the existence of environmental barriers that could affect gene flow between closely related species [7], [21]. Thus, the combination of SDM and phylogeographic analyses is a good strategy to correctly interpret the actual genetic and geographic structure of species.

**Figure 1:**
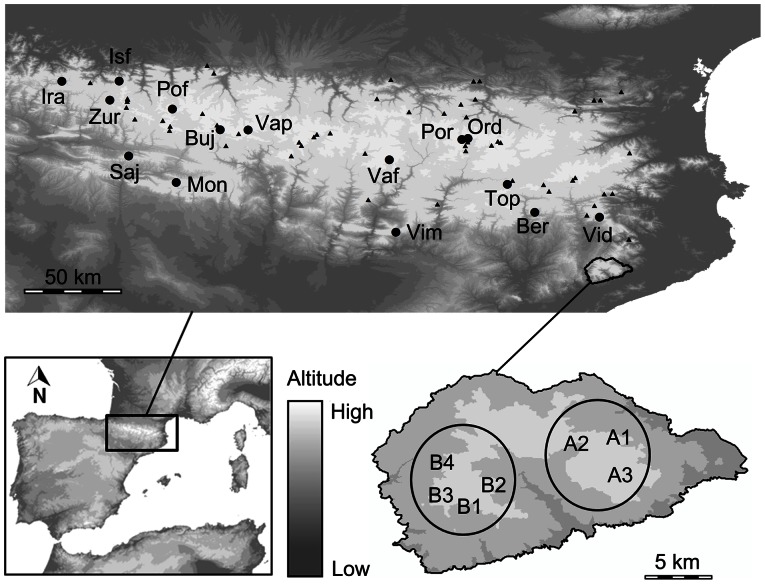
The study area in the NE Iberian Peninsula showing the distribution of the genus *Calotriton*. Circles indicate populations of *C. asper* included in the molecular analyses; triangles correspond to additional populations included in the species distribution modeling (SDM). All localities of *C. arnoldi* are included in the molecular analyses. Locality codes correspond to names on Table 1. Localities of *C. arnoldi* represented do not correspond to the exact geographic locations intentionally due to conservation reasons.

The genus *Calotriton* Gray, 1858, includes only two species adapted to live in cold and fast-flowing waters: the Pyrenean brook newt (*Calotriton asper*) and the Montseny brook newt (*Calotriton arnoldi*). According to Carranza & Amat [20] the two species split during the Pleistocene, approximately 1.1–2 Mya. Although they have been evolving independently, their actual distribution ranges are only separated by 25 km in a straight line. The Pyrenean brook newt is the most widely distributed of the two species, occupying more than 20000 km^2^ across the Pyrenean mountain chain (NE Iberian Peninsula) with some populations extending northwards and southwards, reaching the Prepyrenees [22] (see Figure 1). In contrast, the endemic Montseny brook newt has a very restricted distribution range, occupying a small area of 20 km^2^ restricted to a few brooks in the Montseny massif [23], [24], [25], [26] (see Figure 1). Currently, a total of 7 populations of *C. arnoldi* have been found, fragmented into two main population groups on both sides of the Tordera river valley separated by inhospitable habitat. The eastern and western sectors comprise three and four populations, respectively, with a total estimation of 1000–1500 mature individuals [27]. Owing to its restricted and fragmented distribution and its low population density, *C. arnoldi* is catalogued as Critically Endangered in the International Union for Conservation of Nature (UICN) Red List of Threatened Species. Currently, the conservation planning is in the process of development by the Catalan Government in order to ensure the survival of this species.

The study of the phylogeography of the genus *Calotriton* provides an interesting opportunity to analyze in detail the effects of the Pleistocene climatic changes on the evolution of these two species. Additionally, understanding its population structure and evolutionary history is essential for formulating the appropriate conservation strategies and management proposals, such as identifying the management units (ESU, Evolutionary Significant Unit [28]) for one of the European amphibian species with the smallest distribution range and one of the most endangered. Herein, we use an integrative approach by combining molecular and SDM analyses to unravel the historical processes that have contributed to shaping the biogeography and genetic structure of this south-western Europe endemic newt genus. This work also focuses on the genetic structure of the Montseny brook newt and its morphological differentiation within its small and fragmented distribution range.

## Materials and Methods

### Ethics Statement

The collection of all samples was conducted under the licenses required by the corresponding authorities. Permits were given by the following institutions: Departament d'Agricultura, Ramaderia, Pesca Alimentació i Medi Natural of the Catalan Government, with the permission numbers SF/298 and SF/469 for all samples of *C. arnoldi* and SF/90 and SF/429 for *C. asper*; Servicio de Conservación de la Biodiversidad, Departamento de Desarrollo Rural y Medio Ambiente of the Navarra Government, with the permission number 2012/721 for *C. asper*; Instituto Aragonés de Gestión Ambiental, Area II-Biodiversidad of Aragon Government, with the permission numbers 24/2010/901 and 24/2012/661 for *C. asper*. Tissue samples were collected according to the requirements of the above administrative institutions: newts were captured manually and tissue samples from tail tips and morphological measurements were taken without the use of anesthetics to avoid accidental mortality. Immediately after the completion of the procedure, tissue samples were stored in absolute ethanol and all newts were released at the collection site. No individuals were brought to the laboratory or sacrificed.

### Field sampling and DNA sequencing

A total of 420 individuals were analyzed for the cytochrome *b* gene, of which 315 correspond to *C. asper* from 15 different localities covering most of its distribution range and 105 to *C. arnoldi*, including samples from the complete distribution range of the species (seven populations). For the RAG-1 gene 33 and 75 specimens were analyzed, respectively. The number of individuals collected at each site and sampling locations are reported on Table 1. In order to preserve the critically endangered populations of *C. arnoldi* the three eastern populations are herein refereed as A1, A2, A3, and the four western populations as B1, B2, B3 and B4. Tissue samples consisted of tail tips or fingers preserved in absolute ethanol until further analysis. Genomic DNA was extracted using Qiagen^TM^ (Valencia, California) DNeasy Blood and Tissue Kit following the manufacturer's protocol. A region of 374 bp of the cyt *b* gene was amplified using primers Cytb1EuprF and Cytb2EuprR [20] and using the same PCR conditions as in Carranza *et al*. [29]. Additionally, the following primers were designed to sequence a fragment of 530 bp of the RAG-1 gene: CaloRAG1-831F 5′-CGGTACGAGATCTGGCGGTCC-3′ and CaloRAG1-1362R 5′-TATCTCAGGCACGTGGGCTAGT-3′; the PCR program used for this region included an initial denaturing step at 94°C for 5 min, followed by 35 cycles of denaturation at 94°C for 30 s, annealing at 55°C for 45 s, extension at 72°C for 1 min, and a final extension of 72°C for 5 min. Sequences were aligned using BioEdit 7.0.1 [30] and variable sites were checked visually for accuracy. Coding gene sequences were translated into amino acids using the vertebrate mitochondrial code and standard code, and no stop codons were observed, suggesting that they were probably all functional. Direct sequencing of PCR products for RAG-1 revealed a single nucleotide site at which individuals were heterozygous. Diploid amplified sequences were represented by two alleles [16], [31].

**Table 1:** Sampling locations and number of individuals collected per site (N) for *Calotriton arnoldi* and *C. asper*.

			Mithocondrial features		Nuclear features	
Species	Pop. codes	Sampling site	N	Ocurrence and code (in brackets) of haplotypes	N	Ocurrence and code (in brackets)**of haplotypes
***Calotriton arnoldi***	A1	Montseny, Spain	21	16(h2), 4(h3), 1(h4)	19	26(h2), 12(h3)
	A2	Montseny, Spain	16	16(h2)	9	1(h2), 17(h3)
	A3	Montseny, Spain	5	5(h2)	5	3(h2), 7(h3)
	B1	Montseny, Spain	20	20(h1)	14	28(h1)
	B2	Montseny, Spain	20	20(h1)	10	20(h1)
	B3	Montseny, Spain	20	20(h1)	15	30(h1)
	B4	Montseny, Spain	3	3(h1)	3	6(h1)
		Total *C. arnoldi*	105		75	
***Calotriton asper***	Vid	Vidrà, Spain	15	15(h9)	5	10(h4)
	Ber	Berga, Spain	21	21(h9)		
	Top	La Cerdanya, Spain	19	19(h9)		
	Vim	Vilanova de Meià, Spain	18	18(h9)		
	Ord	Ordino, Andorra	34	4(h5), 30(h9)	1	2(h4)
	Vaf	Vall Fosca, Spain	21	21(h12)		
	Vap	Valle de Pineta, Spain	26	9(h7), 17(h8)		
	Isf	Isaba, France	18	17(h9), 1(h10)		
	Zur	Zuriza, Spain	21	8(h7), 13(h9)		
	Ira	Irati, Spain	15	13(h9), 2(h11)	13	13(h4), 13(h5)
	Mon	Pto. Monrepós, Spain	24	23(h5), 1(h9)	9	18(h5)
	Saj	San Juan de la Peña, Spain	22	18(h5), 4(h6)		
	Por	Port du Rat, France	12	12(h5)		
	Buj	Bujaruelo, Spain	36	33(h5), 3(h8)	5	10(h5)
	Pof	Portalet, France	13	13(h5)		
		Total *C. asper*	315		33	
		Total N	420		108	

Mitochondrial (cytochrome *b*) and nuclear (RAG-1) haplotypes detected in each sampling site (haplotype codes in brackets) and number of occurrences of each haplotype.

### Molecular data analyses

Network approaches may be more effective than classical phylogenetic ones in order to represent intraspecific evolution [32]. As a result networks were inferred using statistical parsimony [33] as implemented in the program TCS 1.21 [34], with 95% connection limit between haplotypes.

An analysis of molecular variance (AMOVA) calculating *F*-statistics [35] was used to determine the level of genetic differentiation between *C. arnoldi* populations from the two different sides of the Tordera river valley (western *vs* eastern populations) using ARLEQUIN ver 3.5.1.2 [36]. Levels of significance were determined through 1023 random permutation replicates. Population genetic structure was assessed by performing the spatial analysis of molecular variance (SAMOVA, version 1.0 [37]). Number of haplotypes (*h*) and variable sites (S), and haplotype (Hd) and nucleotide (Π) diversity indices, for each marker were calculated in DNASP v5 [38].

### Species distribution modelling

*Calotriton arnoldi* was excluded from the SDM as only seven localities are known to date separated each other by a maximum of 6 km. Unfortunately the number of populations is not enough to carry out a reliable prediction. Therefore, SDM was only performed for *C. asper*.

A total of 69 localities (Figure 1), covering the entire distribution range of *C. asper* were used. These localities included our own collection sites (53 localities), and 16 additional species records derived from Milá *et al*.[39].

The study area comprised the northeast part of de Iberian Peninsula (from 44°12′N, 7°18′W to 41°0′N, 3°48′E). Initially, a total of 19 BioClim variables were downloaded from the WorldClim database version 1.4 (http://www.worldclim.org/) to form the present and past (Last Glacial Maximum, LGM and Last Interglacial, LI) climatic datasets [40], [41] at a scale of 30 arc seconds for present and LI periods and 2.5 arc minutes for the LGM. Past climate scenarios for the LGM period were reconstructed by two general atmospheric circulation models: the Community Climate System Model (CCSM, http://www.ccsm.ucar.edu) and the Model Interdisciplinary Research on Climate (MIROC, http://www.ccsr.u-tokyo.ac.jp/~hasumi/MIROC/).

To avoid autocorrelation and over fitting of our data, colinearity among the initial 19 BioClim variables was tested using the Pearson's correlation coefficient in SPSS 17.0 [42], sampling 1000 random points from the studied area ensuring a minimal distance. Seven environmental variables with a Pearson coefficient correlation value lower than 0.8 were retained. The uncorrelated variables that contributed most to the model and therefore were the most likely to influence the occurrence of *C. asper* were selected: Mean Temperature of Driest Quarter (BIO9), Annual Precipitation (BIO12) and Precipitation Seasonality (BIO15). Topogeographical variables and landcover were only available from the same database for current conditions. Altitude was downloaded from WorldClim database, slope was calculated using ARCGIS 10.0 (ESRI, Redlands, CA) and aspect was reclassified into four classes (North, East, South and West) using the same program. Landcover was downloaded from the Global Environment Monitoring database (bioval.jrc.ec.europa.eu). To account for geological range constraints, we additionally used lithology (dominant parent material) derived from the European Soil Database v. 2 [43] as a categorical predictor variable, encompassing 43 categories of geological material in the Iberian Peninsula. Because the geology is unlikely to have changed significantly since the LGM, we treated the lithology as constant [44]. SDM were generated following the maximum entropy modelling implemented in Maxent 3.3.3e [45]. This algorithm has been proven to produce high quality predictions using environmental parameters in combination with geographical presence-data of species , [15], [46], [47]. Geographical occurrences were partitioned between training and test samples (75% and 25%, respectively), as this has been proven to provide high predictive accuracy [48]. The fade-by-clamping option was used in Maxent to remove heavily clamped pixels from the final models. As ensemble model predictions may enhance the reliability and robustness of SDM results [49], a hundred models with randomly selected test samples were computed. The average output probability of presence of the species was set to logistic format. The model performance was evaluated using the area under the curve (AUC) of the receiver operating characteristics (ROC) curve plots, which plot the true-positive rate against the false-positive rate. Models with AUC values above 0.75 are considered useful [46], [50], [51]. The model was evaluated using a null model procedure [52]. We generated 999 sets of 69 random occurrence points (equal to the number of real occurrence points in our dataset) using ENMTools Version 1.3 [53]. We used Maxent to calculate AUC for each of the 999 null datasets and tested whether the AUC of the *C. asper* dataset exceeded the 95^th^ percentile of the null dataset AUCs. These randomly generated models can be used as a null-hypothesis against which to test the significance of species distribution models. If the AUC of the Pyrenean brook newt was significantly higher than the AUC of randomly generated models, it was considered as evidence that the species distribution model performs significantly better than expected by chance [52].

### Morphometric analysis

One hundred and sixty-three adult specimens of *Calotriton arnoldi* were included in the morphological analyses, 57 (36 males and 21 females) and 106 (55 males and 51 females) from western and eastern sectors, respectively. Eight linear morphometric measurements were obtained using a digital caliper (Table 2): snout-vent length (SVL), head length (HL), head width (HW), forelimb length (FLL) and hindlimb length (HLL), limb interval (LI), tail length (TL), and tail height (TH). Sex and adulthood of individuals was determined by the morphology of the cloacal protuberance [20]. Multivariate analyses were used to test geographic differences on morphometric variables between the two sectors within the distribution range of *C. arnoldi* using discriminant canonic analysis and multivariate analysis of variance (MANOVA). All analyses were performed on log-transformed variables using Statistica v.5.5 software (Stat Soft Inc., Tulsa, OK).

**Table 2:** Linear morphometric variables used in the analysis of morphological differentiation of *Calotriton arnoldi*.

Variable	Abbreviation	Linear measurement
Snout-vent length	SVL	From the snout to the posterior margin of cloacal protuberance
Head length	HL	From the snout to the gular fold
Head width	HW	Maximum dorsal head width
Forelimb length	FLL	From the tip of the largest toe to the insertion point at right ventral side
Hindlimb length	HLL	From the tip of the largest toe to the insertion point at right ventral side
Limb interval	LI	Minimum distance between the insertion points at right ventral side
Tail length	TL	From the tip to the posterior margin of the cloacal protuberance
Tail height	TH	Maximum tail height

The pattern of body coloration of 189 *C. arnoldi* (108 from the eastern sector and 81 from the western one) was examined focusing on the lack or presence of pale yellow spots on the dorsal side and patches on the head of the newts.

## Results

### Analyses of genetic structure

**Figure 2:**
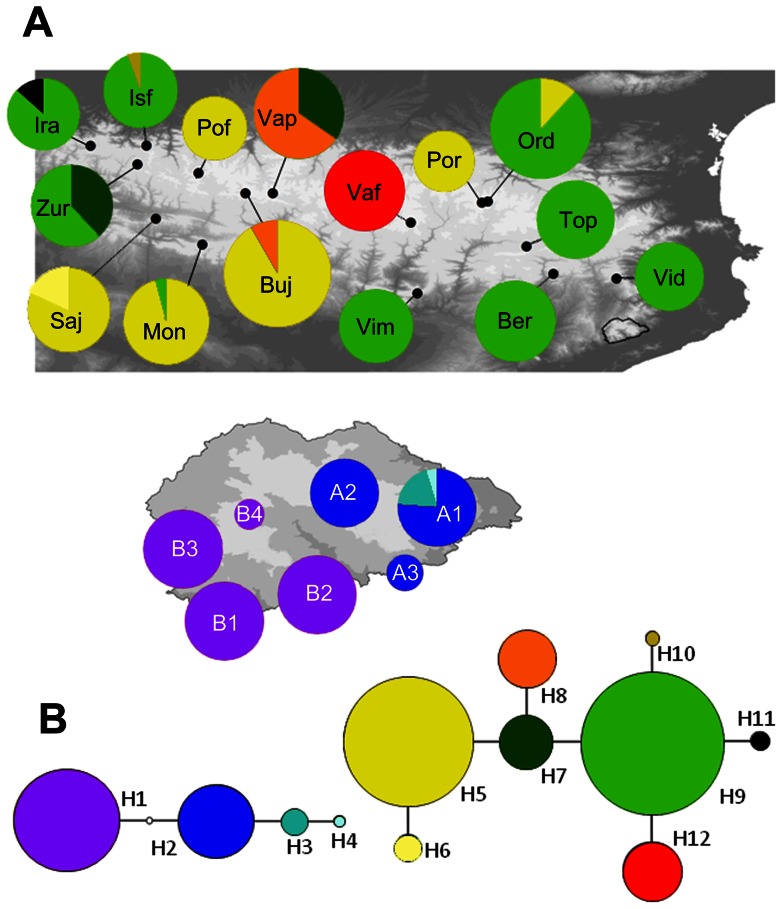
Map showing the geographical distribution of Cyt *b* DNA haplotypes. (A) Pie diagram size indicates number of individuals. (B) Statistical parsimony networks showing cytochrome *b* haplotypes found for *Calotriton arnoldi* (left) and *C. asper* (right); circle size are proportional to haplotype abundance, straight lines and black dots reflect mutations and unsampled or extinct haplotype.

**Figure 3:**
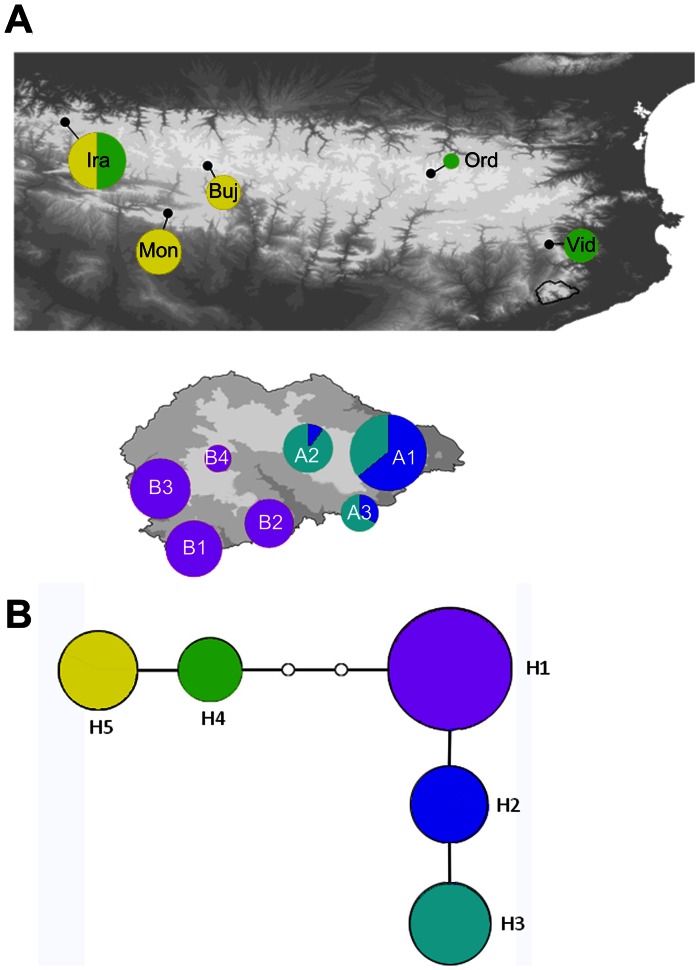
Map showing the geographical distribution of RAG-1 DNA haplotypes. (A) Pie diagram size indicates number of individuals. (B) Statistical parsimony network showing nuclear haplotypes found for *Calotriton* genus; circle sizes are proportional to haplotype abundance, straight lines and black dots reflect mutations and unsampled or extinct haplotype.

The final mtDNA data set included 374 bp of the cyt *b* gene (24 variable and 22 parsimony-informative positions). The nDNA data set included 530 bp (6 variable positions, all of them parsimony-informative). Of the 12 cyt *b* haplotypes identified, 8 were found in *C. asper* and 4 in *C. arnoldi* (Figure 2), whereas of the 5 RAG-1 haplotypes found, 2 corresponded to *C. asper* and 3 to *C. arnoldi* (Figure 3). The number of individuals sequenced and occurrences of each haplotype per sampling site are given in Table 1. The sequences of all mitochondrial and nuclear haplotypes have been deposited in GenBank: accession numbers KC665954–KC665970. The number of haplotypes (*h*) and variable sites (S), and estimates of nucleotide (Π) and haplotype (Hd) diversity for each marker in each sampled species are shown in Table 3. For the mtDNA, similar levels of Π and Hd were observed, being slightly higher for *C. asper* than *C. arnoldi*. Instead, for the nDNA both values were, on average, higher for *C. arnoldi*. Within the Montseny brook newt, all 42 specimens from the western sector corresponded to the same unique haplotype for both mitochondrial and nuclear markers, thus Π and Hd were null.

**Table 3:** Mitochondrial (Cyt *b*) and nuclear (RAG-1) DNA diversity statistics for *Calotriton asper* and *C. arnoldi*.

		*Calotriton asper*	*Calotriton arnoldi*		
		Total	Total	Eastern sector	Western sector
**Cyt ** ***b***	**n**	315	105	42	63
	***H***	8	4	3	1
	**S**	7	4	2	0
	**Π**	0,00331 (0,0001)	0,00276 (0,00019)	0,00066 (0,00026)	0
	**Hd**	0,666 (0,018)	0,495 (0,032)	0,206 (0,076)	0
**RAG-1**	**N**	66	150	66	84
	***H***	2	3	2	1
	**S**	1	2	1	0
	**Π**	0,00090 (0,00006)	0,00163(0,00008)	0,00095(0,00003)	0
	**Hd**	0,478 (0,031)	0,593(0,028)	0,503(0,015)	0

Number of sequences (n), haplotypes (*h*) and variable sites (S), and nuclear (Π) and haplotype diversities (Hd). Standard deviations in brackets.

Geographical distribution of haplotypes and the haplotype networks for cyt *b* and *RAG*-1 markers showing the relationship between both species are displayed in Figures 2 and 3. The statistical parsimony analysis for the mitochondrial marker sorted haplotypes from the two species into independent networks, while all the haplotypes were connected into a single network with the same percentage of divergence for the nuclear marker. The *C. asper* cyt *b* haplotype network comprised eight haplotypes, and adjacent haplotypes were separated by one single step. Individual haplotypes represented samples from between one and eight sampling sites. The *C. arnoldi* haplotype network for the same marker showed four haplotypes. The eastern sector included three haplotypes consecutively connected to each other by one step, and separated from the single western haplotype by two steps. The single RAG-1 parsimony network showed three steps between *C. arnoldi* and *C. asper* haplotypes, while one step connected haplotypes within each species.

Analyses of molecular variance were consistent with high levels of structure in both markers, when populations of the genus *Calotriton* were divided into species groups (Table 4). For the cyt *b* marker, the overall differentiation among populations was high and statistically significant (F_ST_  = 0.988, *P*<0.001), and for the nuclear marker, the overall F_ST_ value was 0.960 (*P*<0.001).

**Table 4:** Analysis of molecular variance (AMOVA) between *Calotriton asper* and *C. arnoldi*.

	Percentatge of variation			Fixation indices		
	Among groups	Among populations within groups	Within populations	F_SC_	F_ST_	F_CT_
**Cyt ** ***b***	91.44	7.38	1.19	0.86111**	0.98810**	0.91435**
**RAG-1**	79.61	16.40	3.99	0.80429**	0.96010**	0.79611*

* P<0.05, ** P<0.0001.

Within *C. arnoldi*, the genetic structure obtained by SAMOVA identified the same geographic structure found among the haplotype networks drawn in the phylogeographic analyses. The best partitioning of the genetic diversity by SAMOVA was obtained when samples were grouped into two groups, corresponding to the western and eastern sectors (data shown in Table 5 for *K* = 2). The genetic structure found in AMOVA analyses for the mitochondrial marker showed an overall F_ST_  = 0.954 (*P*<0.0001). Among groups variation was statistically significant (F_CT_  = 0.949; *P*<0.05). Only 0.52% of variance was a result of differences among populations within these groups, and 4.59% within populations. The nuclear marker showed similar but slightly lower values (F_ST_  = 0.896, *P*<0.0001; F_CT_  = 0.832; *P*<0.05). Most variation was explained by the among groups differences (83.16%), with variation values of 6.45% and 10.39% for among-populations within-groups and within-populations, respectively.

**Table 5:** Spatial analysis of molecular variance (SAMOVA) and analysis of molecular variance (AMOVA) for *Calotriton arnoldi*.

		Percentatge of variation			Fixation indices		
		Among groups	Among populations within groups	Within populations	F_SC_	F_ST_	F_CT_
**SAMOVA**	**Cyt ** ***b***	93.23	0.48	6.28	0.07149	0.93716**	0.93716*
	**RAG-1**	83.20	5.46	11.34	0.32489**	0.88659**	0.83201*
**AMOVA**	**Cyt ** ***b***	94.89	0.52	4.59	0.10136	0.95410**	0.94892*
	**RAG-1**	83.16	6.45	10.39	0.38313**	0.89609**	0.83156*

* P<0.05, ** P<0.0001

Two geographical groupings are showed corresponding to eastern and western sectors.

### Species distribution modelling

**Figure 4:**
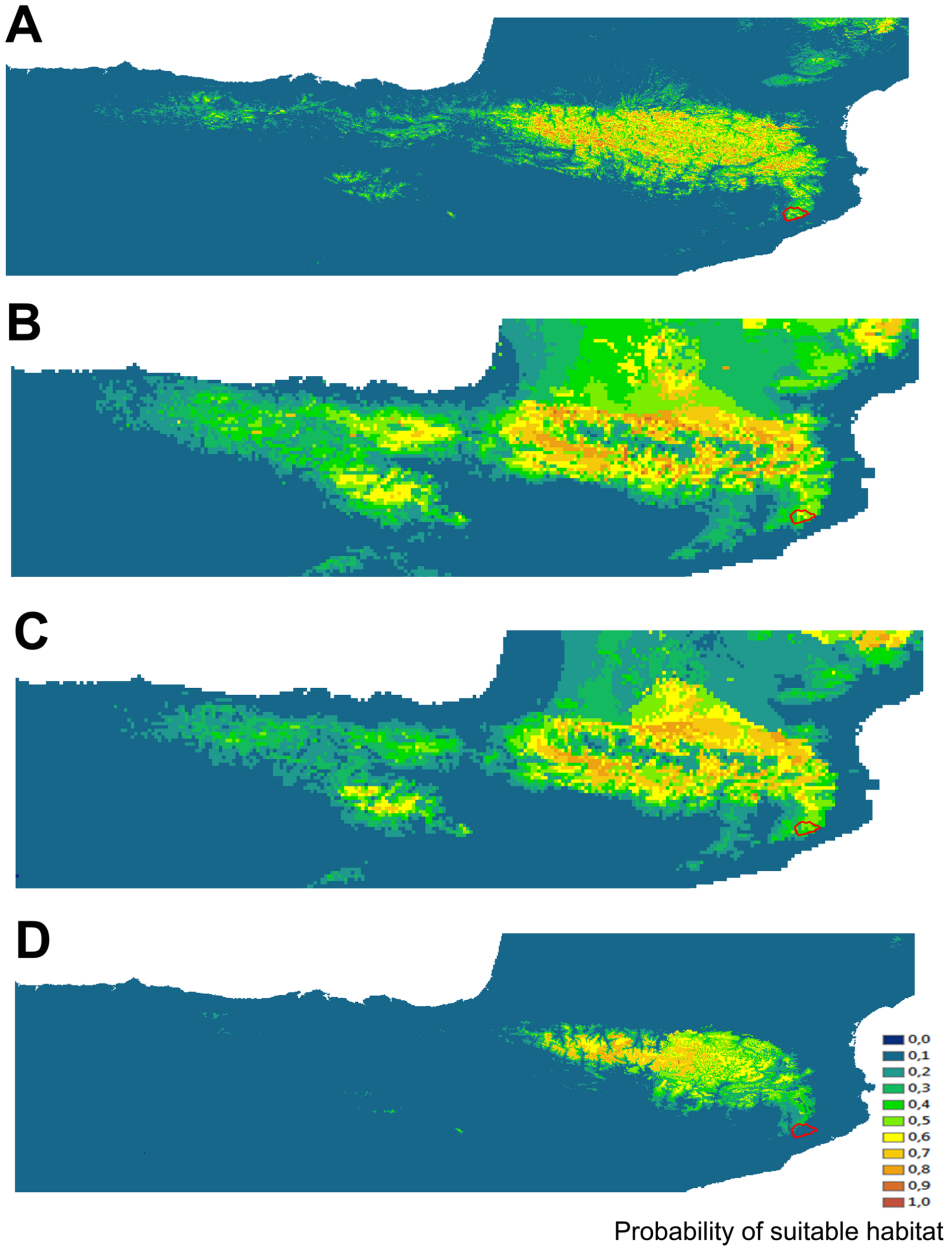
Predicted distribution models of *Calotriton asper*. (A) Present distribution using climate and topographic variables, landcover and lithology. Last Glacial Maximum based on (B) CCSM model and (C) MIROC model, and (D) Last Interglacial distributions using climate variables and lithology. Warmer colors represent areas of high habitat suitability. Red marks and arrows indicate the Montseny Mountain Natural Park.

The predicted geographic distributions for *C. asper* under present and past conditions are shown in Figure 4. Maximum entropy modelling produced high predictive accuracy models, according to the average testing AUC for the present SDM using climate, topography, lithology and landcover variables (AUC  = 0.934±0.013). SDM inferred for the past conditions using climate and lithology also showed overall an adequate fit to the distributions. The distribution models based on the LI conditions presented an AUC value of 0.921±0.016, and over 0.92 based on LGM predictions (CCSM 0.924±0.016, MIROC 0.925±0.013). The AUC of these models were significantly higher than the null-model AUCs (the 95^th^ percentiles of the null dataset were 0.618, 0.620, 0.629 and 0.619 for the present, CCSM, MIROC and LI, respectively). This indicates a good fit of the models.

SDM predictions based on current climate data showed a wide predicted distribution throughout the Pyrenean mountain chain (NE Iberian Peninsula), with some areas of suitable habitat southwards along the Montseny massif (Figure 4), although a slight disruption of the distribution can be observed when the connection area between the Pyrenees and Montseny is focused. The projection of this model onto Pleistocene climate surfaces indicates different periods of expansion and retraction. The predicted distribution based on LI conditions indicates lower surface of suitable habitat in comparison to the present predicted distributions. This model showed that the suitable distribution for *C. asper* during the last interglacial was restricted to the central areas of the Pyrenees, being almost absent southwards of the Montseny. During the last glacial maximum *C. asper* presented a range expansion. The predicted distribution spreads to lower altitudes, being absent from the highest parts of the mountain chain. A continuous predicted distribution from the Pyrenean chain to the southern Montseny massif is observed. The extent of range expansion is variable depending on the climatic model, being more apparent under the CCSM than under the MIROC. These models indicate that several areas of suitable habitat (potential glacial refuge) existed in the glacial periods.

### Morphological data

**Figure 5:**
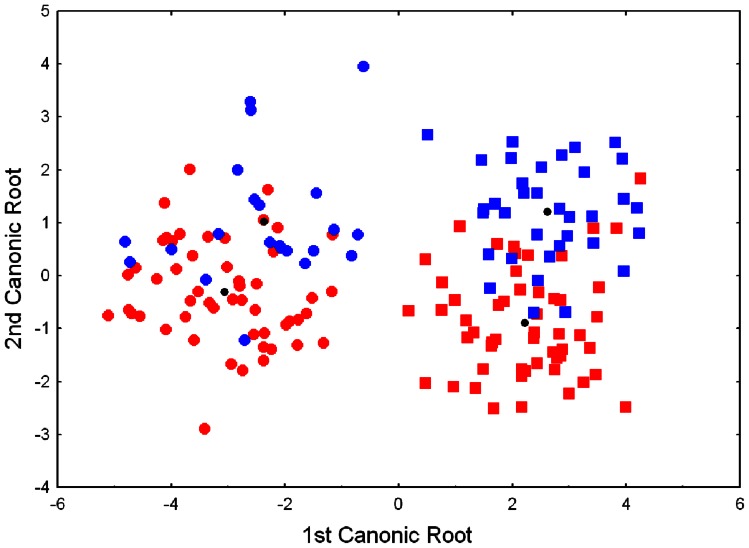
Discriminant canonical analysis scatterplot of males and females of *Calotriton arnoldi* from eastern and western sectors. Red squares and circles indicate eastern males and females, and blue squares and circles show western males and females, respectively. Black points are the mean values for each group.

MANOVA analysis found sexual (Wilk's Lambda _8 152_ = 0.143; *P*<0.0001) and population (Wilk's Lambda _8 152_ = 0.576; *P*<0.0001) significant differences without interaction between these two factors (Wilk's Lambda _8 152_ = 0.907; *P* = 0.058). A posthoc test for unequal size showed no significant differences among sexes or populations in SVL and LI while HL and TH were the only sexually discriminant variables (Table 6). The length of the two limbs, HW (with the only exception of the comparison between western and eastern females) and TL (with the only exception of the comparison between western females and eastern males) indicated population and sexual differences. The Discriminant analysis showed sexual separation based on the first canonic root (Figure 5): males have short and high tails, in comparison with females. Population differences were less marked and exclusively defined by the second canonic root. Males and females from eastern populations have longer limbs than western ones, while males from western populations have wider heads and longer tails than eastern ones.

**Table 6:** Weight coefficients of eight linear morphometric measurements for the two first canonic from discriminant analysis.

Variable	1st Canonic Root	2nd Canonic Root
SVL	−0.369	0.504
Head length	0.128	−0.395
Head width	0.144	0.541
Forelimb length	0.002	−0.372
Hindlimb length	0.414	−0.167
Limb interval	0.054	0.109
Tail length	−0.465	−0.881
Tail high	0.907	−0.109
Eigenvalues	7.024	0.762
% Cummulative proportion of variability	89.4	99.1

Eigenvalues and percentage of accumulated variance are listed.

**Figure 6:**
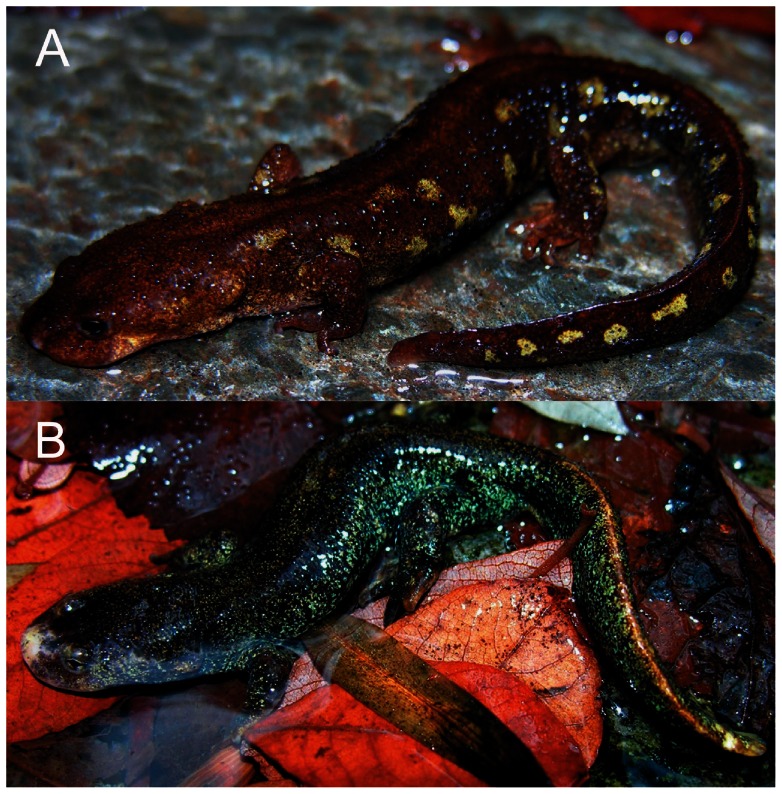
Characteristic patterns of coloration of newts belonging to the two sectors of *Calotriton arnoldi* range. (A) Adult female from the eastern populations showing yellow spots found in 78.8% of the individuals, and (B) adult male from the western populations with the whitish margin of the snout.

Our analyses of coloration found striking differences between sectors defined by two traits: dorsal yellow spots and whitish margin of the snout (Figure 6). The first trait was never found in western populations (Chi-square test X^2^_1_ = 106.312, *P*<0.001) and therefore is exclusive of the eastern populations. The coloration of the margin of snout is a new diagnostic character that is always present in males of the western sector, was found only in a female of the western sector, and it has never been found in male or female *C. arnoldi* of the eastern sector (Chi-square test X^2^_1_ = 67.652, *P*<0.001).

## Discussion

### Evolutionary history of the genus *Calotriton*

Our study indicates that there is an unexpectedly high level of lineage sorting between the two *Calotriton* species and within *C. arnoldi*, both with nuclear and mitochondrial genes. Despite being phylogenetically very closely related [20], the network analysis of the nuclear gene fragment RAG-1 (Figure 3) shows that all 66 alleles of *C. asper* and all 150 alleles of *C. arnoldi* analyzed in the present study are unique for each species. Moreover, the analysis of all 315 mtDNA sequences of the cyt *b* gene of *C. asper* and all 105 sequences of *C. arnoldi* resulted in two independent networks with all sequences being unique for each species (Figure 2). These results indicate that, despite their geographic proximity and morphological similarity, there is no evidence of gene flow between these two species, i.e. there is no signal of genetic introgression between them. This is especially relevant taking into account the short geography distance between the closest populations of each species (less than 25 km), the overall habitat similarity and the same courtship behavior based on female caudal capture [54]. Strong intraspecific genetic structuring and interspecific divergence is found in other salamanders including situations of sympatry, allopatry and parapatry in small mountain ranges with limited or complete absence of gene flow (see for example the salamanders of the *Plethodon ouachitae* complex [12], [55], [56]). The differences in the degree of genetic differentiation found between RAG-1 and cyt *b* are not surprising, especially if one takes into account that population history produces less phylogeographic signal with nuclear than with mitochondrial DNA data owing to their established divergence rates [15], [57], [58]. The results of the AMOVA confirm this noteworthy specific differentiation being statistically significant for both markers and, as above, more notable in the mitochondrial than in the nuclear data (with variances among species of over 90% and near 80%, respectively) (Table 4). This differentiation suggests that *Calotriton* species have not been in contact for a long time. These results are in accordance with the speciation time determined for these species [20], which occurred during the Pleistocene, around 1.1–2 Mya.

As shown in Figure 4, suitable climatic conditions for *C. asper* seem to have been present in the Montseny massif without a prominent barrier, implying a possible contact zone at present and during the LGM. These results contrast with the genetic findings, as no signal of hybridization has been found between the two species. Moreover, *C. asper* has never been reported in the Montseny massif, which is occupied by *C. arnoldi* [20]. Therefore, although they seem to share a common potential niche, these species are completely isolated and their distributions appear to be disjoint. Thus, our results suggest that these sister species originated in allopatry during sharp Pleistocene glaciations, when some populations probably found refuge in the Pyrenean axial chain and others in the Montseny massif [20]. Secondarily, the species' potential distributions may exhibit spatial overlap with each other due to their present niche similarity [7]. Factors such as predators (e.g. brown trout [59]) or lack of time for secondary contact following vicariance [7] may explain why the *C. asper* population expansions did not reach the Montseny massif. According to these findings, despite that the actual predictive distribution of *C. asper* covers the Montseny massif, we were unable to detect any current or past hybridization events between these two species.

Regarding the SDM results and some fossil records from Cova Colomera (Montsec, Lleida) [60], it is suggested that *C. asper* has survived across the Pyrenean axial mountains, avoiding the highest altitudes during periods of sharp glacial climatic conditions (LGM). Paleoclimatic studies have detected periglacial events during the last glaciations [61], indicating alpine condition in the ridges of the Montseny separating the south (the Tordera Valley where *C*. *arnoldi* is distributed), from the north face of the massif. It is possible that these harsh environmental conditions could have prevented the expansion of the Pyrenean species to the Montseny during the LGM period. Climatic conditions during LI were in many aspects similar to current ones in the Western Mediterranean basin ([62] and references therein), thus it is not surprising the similarity found between LI and present SDM maps. The fact that *C. asper* can be found at high altitude [20] may explain that during warmer periods (LI and present) the species disappeared from lower altitudes. During the warmer periods, the distribution of this species has been confined to the Pyrenean and pre-Pyrenean mountains [63] (Figure 4). Although the Pyrenean newt is a highly polymorphic species [63], [64], [65], [66], the genetic results (only 8 and 2 haplotypes found for cyt *b* and RAG-1, respectively across more than 20000 km^2^) suggest that this widespread species presents a low level of genetic variability in the two genes analyzed here compared to its congener, the Montseny brook newt, which presents an extremely small distribution range (20 Km^2^). Morphological differences may appear under local selection pressure in response to population specific ecological conditions [66]. These results indicate that not enough time has passed yet to find these phenotypic differences fixed into the genotype of the morphologically different populations of *C. asper*. The low genetic variability of *C. asper* may be explained by the much broader climatic suitability shown during the LGM that could allow the connection of populations and subsequent homogenization as a consequence of gene flow [46] and the subsequent recent re-colonization of the highest altitudes not long ago after the last glacial maximum [20], [67]. A similar pattern of low level of genetic variability is found in *Rana pyrenaica* [67], a species that shares geographical distribution and potential niche (mountain streams) with *C. asper*. Even though, owing to the discrepancies of high levels of variability found with AFLPs markers [39], further studies using faster markers like microsatellites are required to better understand its population structure.

### Population structure within *Calotriton arnoldi*

Despite having been described in 2005 and its relevance from a conservation and evolutionary point of view, this is the first attempt to assess the level of genetic and morphological differentiation of *C. arnoldi*.

Here, the nucleotide and haplotype diversities have been analyzed to infer the genetic structure of the Montseny brook newt and the level of gene flow among populations on each side of the Tordera river valley. Our data show that despite having a distribution range 1000 times smaller than *C. asper*, the Montseny brook newt presents a similar level of genetic variability in the mtDNA and a higher level of genetic variability in the nDNA (Figures 2 and 3; Table 3). Whereas the mitochondrial data shown above for *C. asper* and Milá *et al*. [39] accounted for eight and five different haplotypes, respectively covering a distribution area of over 20000 km^2^, the Montseny brook newt presents four different haplotypes but in less than 20 km^2^. One of the possible explanations of these genetic differences between these two sisters species is that *Calotriton asper* has a higher dispersion capacity than *C. arnoldi*. The juveniles of the Pyrenean brook newt present a terrestrial phase that starts after metamorphosis and can last up to two years before they return definitively to the adult aquatic lifestyle. During this terrestrial period, juveniles change morphology and can disperse away from the stream in which they were born, contributing to the gene flow among populations [68]. Contrary to *C. asper*, it has been shown that the Montseny brook newt is completely dependent on water during its whole life, as none of the different life stages (from larvae to adult) have been found in land [20], [69]. Other adaptations to an exclusive aquatic lifestyle include extremely reduced lungs to reduce buoyancy and very thin and smooth skin (transparent underneath) to facilitate cutaneous respiration [20], [69]. This species is restricted to cold and well-oxygenated mountain streams. Therefore, it is unable to exchange individuals through a terrestrial environment or aquatic environments of unsuitable habitat (with waters above 15°C), which favors population isolation. The Sardinian brook newt, *Euproctus platycephalus*, a species that inhabits similar environments as *C. arnoldi*, presents a high genetic structure [70]. The authors suggest that effective population sizes may be large enough to allow such a complex diversification in a small distribution area. In our case, *C. arnoldi* not only presents a much smaller distribution range than *E. platycephalus*, but also its effective population size could be much smaller [27].

In general, the phylogeographic analyses of *C. arnoldi* are concordant with the geographical structure within its distribution range. Our data show a strong genetic differentiation at both the mitochondrial and nuclear levels between eastern (A1–A3) and western (B1–B4) populations of *C. arnoldi* (Figures 1, 2 and 3; Table 5). The haplotype networks suggest a high degree of isolation between western and eastern populations, as all 63 cyt *b* and 84 RAG-1 sequences of the western populations and all 42 cyt *b* and 66 RAG-1 sequences of the eastern populations are unique for each geographical area (Figures 1, 2 and 3; Table 1). This striking level of isolation across such a small geographic distance is particularly clear and well supported by the results of the AMOVA and SAMOVA (Table 5). Despite the distance between the two population sectors is just around 6 km in a straight line, at present there is no possible connection between the western and eastern populations across suitable habitat. Even though they may be connected following the watercourse of the hydrographical basin, a distance of over 60 km should be covered. Moreover, the watercourse does not provide an adequate environment, due in part as a result of the characteristics of the stream but also because it descends below the 600 m, where water becomes too warm for the long-term survival of *C. arnoldi* [25].

In accordance with the results of the molecular analyses (see above), population distribution is a significant factor of morphological differentiation in *C. arnoldi*. MANOVA, discrimination analysis based on morphometric data, and patterns of coloration indicates a clear differentiation between the western and eastern populations, reinforcing their mutually isolation.

### Implications for the conservation of *C. arnoldi*

One of the major objectives of any conservation plan is the maintenance of genetic variability [71]. The results obtained in this study will certainly be helpful for the conservation program of the Montseny brook newt. Habitat disturbance including large amounts of water extracted for commercial purposes, deforestation and the existence of tracks and roads that disrupt the brook continuity, are all major threats affecting this species [25], [26], [72] similarly to other isolated glacial amphibian relicts [73]. As a result, the Montseny is changing and drying out at a considerable pace and the distribution of *C. arnoldi* is being reduced dramatically [27]. The low population size of the species together with its reduced and disturbed distribution area requires urgent management guidelines for the long-term survival of this species.

Population genetics is a useful tool for the planning and development of conservation programs [16], [70], [67]. The level of genetic structuring in *C. arnoldi* shown here would justify the conservation of two distinct management units (ESU, Evolutionary Significant Units [28]). To date, one of the management measures for the conservation of this species is the development of a captive breeding program, which started in 2007 by the Catalan Government. Based on biogeography, morphology and preliminary genetic data, the breeding program maintained individuals from both sectors separately into two ESU's. The present results confirm the need for continuing with the maintenance of two breeding stocks. Genetic data has played and will play an important role in the conservation of the Montseny brook newt, as for instance in guiding any introduction or translocation of individuals in order to maintain the genetic variation, and thus, guarantee the long-term viability of the populations [74].

Further genetic studies using microsatellites markers are in course to infer the current gene flow among populations and their possible isolation within sectors. This is of vital importance in order to establish whether further ESU's within sectors should be defined.
